# Room Temperature Crystallized Phase‐Pure α‐FAPbI_3_ Perovskite with In‐Situ Grain‐Boundary Passivation

**DOI:** 10.1002/advs.202400275

**Published:** 2024-03-19

**Authors:** Zejiao Shi, Yaxin Wang, Yanyan Wang, Xiaoguo Li, Xiaofei Yue, Haoliang Wang, Xin Zhang, Liangliang Deng, Chongyuan Li, Jiao Wang, Zuoti Xie, Yinguo Yang, Chunxiao Cong, Anran Yu, Yiqiang Zhan

**Affiliations:** ^1^ Center for Micro Nano Systems School of Information Science and Technology (SIST) Fudan University Shanghai 200433 P. R. China; ^2^ Department of Materials Science and Engineering MATEC Guangdong Technion – Israel Institute of Technology Shantou Guangdong 515063 P. R. China; ^3^ School of Microelectronics Fudan University Shanghai 200433 P. R. China

**Keywords:** crystallization, FAPbI3 perovskite, grain boundary passivation, phase transition

## Abstract

Energy loss in perovskite grain boundaries (GBs) is a primary limitation toward high‐efficiency perovskite solar cells (PSCs). Two critical strategies to address this issue are high‐quality crystallization and passivation of GBs. However, the established methods are generally carried out discretely due to the complicated mechanisms of grain growth and defect formation. In this study, a combined method is proposed by introducing 3,4,5‐Trifluoroaniline iodide (TFAI) into the perovskite precursor. The TFAI triggers the union of nano‐sized colloids into microclusters and facilitates the complete phase transition of α‐FAPbI_3_ at room temperature. The controlled chemical reactivity and strong steric hindrance effect enable the fixed location of TFAI and suppress defects at GBs. This combination of well‐crystallized perovskite grains and effectively passivated GBs leads to an improvement in the open circuit voltage (*V_oc_*) of PSCs from 1.08 V to 1.17 V, which is one of the highest recorded *V_oc_* without interface modification. The TFAI‐incorporated device achieved a champion PCE of 24.81%. The device maintained a steady power output near its maximum power output point, showing almost no decay over 280 h testing without pre‐processing.

## Graphical abstract

A combined method is proposed by introducing 3,4,5‐Trifluoroaniline iodide (TFAI) into the perovskite precursor. The TFAI induces a complete phase transition of α‐FAPbI_3_ at room temperature and serves as a passivation agent at grain boundaries. The TFAI‐modified device achieves over 24% PCE and maintains steady power output for 380 h with almost no performance decay.

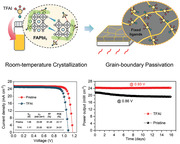

## 1 Introduction

Organic‐inorganic hybrid perovskite has emerged as a next‐generation photovoltaic (PV) material due to its unique power conversion properties.^[^ ^1^, ^2^, ^3^ ^]^ The certificated power conversion efficiency (PCE) has recently attained up to 26.1%.^[^ ^4^ ^]^ The cost‐effective and energy‐saving fabrication of perovskite solar cells (PSCs) has attracted much attention toward upscaling applications.^[^ ^5^, ^6^, ^7^ ^]^

The black phase formamidinium lead triiodide (α‐FAPbI_3_) is one of the most promising perovskite materials with a suitable optical band gap of 1.52 eV.^[^ ^8^ ^]^ However, FAPbI_3_ perovskite suffers from two critical issues the phase transition and the grain boundaries (GBs) defects.^[^ ^9^, ^10^, ^11^ ^]^ δ‐FAPbI_3_ which is an undesirable photoinactive perovskite phase has a stable structure at room temperature due to its low formation energy and compact crystallization.^[^ ^12^ ^]^ Therefore, the FAPbI_3_ perovskite generally possesses poor crystallinity even after annealing at high temperature.^[^ ^13^ ^]^ Besides, the energy loss at grain boundaries (GBs) where the trap density is 2 orders of magnitude higher than that of in the bulk is another issue of concern to community. Accumulated halide deficiency and formamidinium vacancy at GBs tend to form the sub‐band states^[^ ^14^, ^15^ ^]^ resulting in ion migration, electrostatic potential barrier, and lattice deconstruction and making the device sensitive to the boundary‐localized traps.^[^ ^15^, ^16^, ^17^ ^]^

Many pioneer works regarding the crystallization regulation or GBs passivation have been conducted, respectively. In terms of grain manipulation, Research on additives, template growth, and post‐treatment have been devoted. Additives, which serve as dopants or crystallization agents are popular for obtaining well‐crystallized perovskite films. For instance, methylammonium chloride (MACl), a Cl‐based additive, has been reported to aid in the formation of intermediates with FAPbI_3_ and increase grain size.^[^ ^18^ ^]^ Engineered tin oxide (SnO_2_)^[^ ^19^ ^]^ and nickel oxide (NiO_x_)^[^ ^20^ ^]^ also act as interlayers that serve as templates for FAPbI_3_ growth. Lu et al. developed a vapor‐assisted deposition method for post‐treatment by introducing methylammonium thiocyanate vapor treatment to convert the δ‐FAPbI_3_ to α‐FAPbI_3_ at low temperature.^[^ ^13^ ^]^

In terms of GBs passivation, Efforts have been devoted to Ruddlesden‐Popper (RP) perovskite engineering,^[^ ^21^, ^22^, ^23^ ^]^ 2D/3D heterojunction binding^[^ ^24^, ^25^, ^26^ ^]^ and interface modification.^[^ ^27^, ^28^, ^29^ ^]^ Quasi‐2D perovskite tailors the 3D structure into 2D fragments to prevent undesired vacancy defects. The interaction between the cations and [PbI_6_]^4−^ at GBs are reinforced by properly selected space cations.^[^ ^30^, ^31^, ^32^, ^33^ ^]^ Recently, Both the top^[^ ^34^ ^]^ and the bottom^[^ ^35^ ^]^ formed 2D/3D heterojunction perovskite has been found to benefit GBs passivation due to the decreased interface resistance and manipulated photochemical degradation. A generally applied GBs modulation strategy toward state‐of‐the‐art PSCs with record PCEs^[^ ^36^, ^37^ ^]^ involves interface modification materials with amino terminals or other electron donor groups serving as Lewis bases and bonding with the perovskite components vacancies.

However, the above‐mentioned strategies with respect to grain manipulation and passivation of GBs are often carried out separately due to the complicated crystallization and defect formation mechanisms. Therefore, there is an urgent need for a method that combines both crystallization regulation and GBs passivation simultaneously. This work presents a method for controlled crystallization of perovskite by passivating grain boundaries through the introduction of 3,4,5‐trifluoroaniline iodide (TFAI) as an engineered bonding ligand into the precursor. TFAI has a lower affinity to [PbI_6_]^4−^ than DMSO, which prevents the generation of coordinated products and allows for the formation of united micro‐sized clusters in the precursor. It has been confirmed that the incorporation of TFAI is favorable for the complete perovskite phase transition to α‐FAPbI_3_ at room temperature. It is noted that strong steric hindrance spontaneously fixes the TFAI at perovskite grain boundaries. The interaction is manipulated through Pb‐F chelation and FA^+^‐TFA^+^ bonding, which effectively passivates film defects and suppresses leakage current at grain boundaries. We have fabricated the n‐i‐p structure PSCs with and without TFAI modification, where the perovskite layer is sandwiched between the electron transport layer (ETL) of SnO_2_ (at the bottom of the perovskite) and the hole transport layer (HTL) of Spiro‐OMeTAD (on the top of the perovskite). The incident light from the FTO/SnO_2_ side is captured in the perovskite layer. The photogenerated electrons move toward FTO anode, while the holes move toward the Au cathode. The energy alignment diagram supports the well‐regulated charge transport in the TFAI‐modified device. As a result, we achieved a champion PCE of up to 24.81%, mainly due to an improvement in *V_o_*_c_ from 1.08 V to 1.17 V. This is one of the highest recorded *V_oc_* values without interface modification. The unencapsulated devices maintain a steady power output under standard AM 1.5G irradiation for over 380 h, maintaining almost 100% of their initial efficiency.

## 2 Results and Discussion

### 2.1 Regulated Crystallization of α‐FAPbI_3_ Film

Scanning electron microscopy (SEM) was first performed to study the perovskite morphology before and after incorporating TFAI into the precursor. The perovskite films containing 0% (denoted as pristine), 1% (denoted as TFAI 1%), and 5% TFAI (denoted as TFAI 5%) are prepared by the method involving FAI, PbI_2_, MACl, and MDACl_2_. Figure S1a–c (Supporting Information) shows the perovskite surface morphology with different TFAI concentrations. The TFAI 1% film has an enlarged average grain size of 1.96 µm compared with the pristine of 1.29 µm (Figure S2, Supporting Information). It's found that white PbI_2_ nano sheets arise at the perovskite GBs on the pristine film. Our previous study has shown that the remaining PbI_2_ mainly comes from the decomposed perovskite film after annealing.^[^ ^38^ ^]^ It's also confirmed by the XRD results of as‐deposited films before annealing, which will be discussed later. Upon adding 1% TFAI, the GBs show the suppressed PbI_2_ phase. According to Park et al., TFAI cannot form the 2D perovskite structure due to its low reactivity with bulk perovskite. However, it acts as a bonding ligand at the interface between perovskite and TFAI capping layer,^[^ ^27^ ^]^ thereby preventing the appearance of PbI_2_. By maintaining a concentration up to 5%, the white phases might be related to the PbI_2_^[^ ^39^ ^]^ or non‐perovskite complexes after annealing^[^ ^29^, ^40^ ^]^ begin to orient themselves on the surface of the perovskite. The overloading TFAI has a impact on the perovskite crystallization process. It is suspected that the lower affinity of residual PbI_2_ or produced non‐perovskite complexes to well‐passivated GBs repels the components to the top of the film and causes the surface distribution. while the double‐side effects of remaining PbI_2_ or non‐perovskite complexes are still debatable,^[^ ^39^, ^41^ ^]^ it has been confirmed that a well‐controlled phase distribution can improve the open circuit voltage (*V_oc_*) by reducing the vacancy defects.^[^ ^42^, ^43^ ^]^

**Figure 1:**
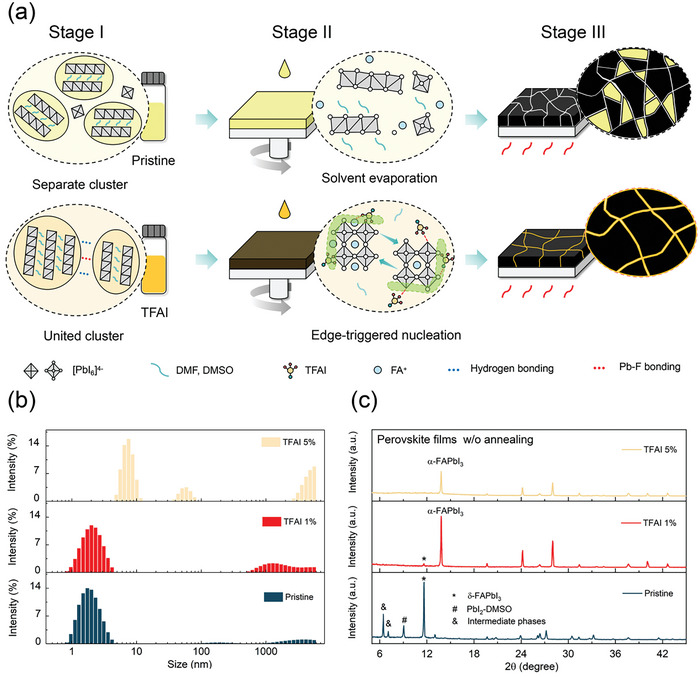
Perovskite crystallization. a) Schematic diagram of perovskite nucleation and crystallization w/o and with 3,4,5‐Trifluoroaniline iodide (TFAI). It processes three stages involving precursor cluster aggregation (stage I), anti‐solvent assisted nucleation (stage II), and annealing grain growth (stage III). b) Dynamic light scattering (DLS) measurement of pristine and TFAI‐incorporated precursors. c) XRD patterns of as‐deposited pristine and TFAI film before annealing. * represents δ‐FAPbI_3_, # represents PbI_2_‐DMSO complex and & represent intermediate phases.

The process of forming a perovskite film typically involves three stages involving precursor preparation (stage I), spin coating (stage II), and post annealing (stage III) as shown in **Figure** **1**a. At stage I, the perovskite precursor is prepared, resulting in the formation of nano‐sized clusters due to the strong coordination interaction between PbI_2_ and DMF/DMSO solvent, as shown at the top of Figure 1a and in Figure 1b. After the anti‐solvent is dropped at stage II, the solvent is rapidly drained during spin‐coating leaving the semi‐dried mix phases of δ‐FAPbI_3_, intermediate phases, and the PbI_2_‐DMSO complex (Figure 1c). The as‐deposited film is then heated to initiate phase transition and crystallization at stage III. In the pristine sample, It is suggested that the heterogeneous nucleation is dominated as it occurs mainly at the phase boundary and the film surface, where the lowest bond formation energy is allowed.^[^ ^44^, ^45^ ^]^ With prolonged annealing time, unevenly grown GBs and degraded production of PbI_2_ are distributed throughout the sample after crystallization.

The precursor incorporated with TFAI becomes darker with increasing amounts of TFAI, as shown in Figure S3 (Supporting Information). Dynamic light scattering (DLS) results indicate that the nano‐sized clusters enlarge into micron‐sized unions as shown in Figure 1b. This increase in size is attributed to variations in coordination and hydrogen bonding.^[^ ^46^, ^47^ ^]^ In general, strong coordination restricts colloid expansion and tends to form intermediate phases in the precursor.^[^ ^48^ ^]^ However, in our case, we confirmed that the interaction between TFAI and Pb^2+^ is weaker than that of DMSO, as shown by electrostatic surface potential (ESP) calculation (Figures S4 and S5, Supporting Information). Therefore, we suggest that the TFAI has limited effect within each individual cluster, but serves as the union agent between the formed clusters at stage I as shown at the bottom of Figure 1a. The clusters that are aggregated are beneficial for reducing nucleation sites and improving nucleation.^[^ ^49^ ^]^

Compared to the pristine sample, the orange film quickly turns dark brown after spin coating at stage II, indicating the phase transition occurred. We conducted X‐ray diffraction (XRD) measurements to check the as‐deposited film before annealing (Figure 1c). The pristine sample possesses clear signals at 9.1° and 11.6° with respect to the PbI_2_‐DMSO complex and δ‐FAPbI_3_, while no PbI_2_ peak is observed. In this semi‐dry film (as‐deposited before annealing), some intermediate phases are also identified at small angles of the pristine sample. The TFAI 1% sample shows a strong signal of α‐FAPbI_3_, while a very weak peak of δ‐FAPbI_3_ is observed. The δ‐FAPbI_3_ phase further disappears or below the detection limitation of the instrument, as the TFAI is increased up to 5% in the perovskite film. Hence, we propose the phase‐transition mechanism at stage II. After partially removing the Pb^2+^ coordinated solvent DMF/DMSO, the united cluster in the TFAI sample tends to produce fluoride‐terminated [PbI_6‐x_F_x_]^4−^ frameworks in the semi‐dry film, which is favorable for the subsequent nucleation and crystallization. First, the TFAI constructs the fluoride‐terminated nucleus surface, as the formation energy of Pb‐F (−1.99 eV atom^−1^) is much lower than that of the Pb‐I (−0.95 eV atom^−1^).^[^ ^50^, ^51^ ^]^ The bonding energy evaluation of Pb‐F and Pb‐I was conducted to reveal the capability of bond transformation as described in Note S1 (Supporting Information).

The formation energy of *E* is denoted as the energy released when free ions bond together. The $E_{\left(PbF_{4}\right)}$ in tetragonal structure is estimated to be ‐5056 kJ mol^−1^ which is more negative than that of the $E_{\left(PbI_{2}\right)}$ (‐1806 kJ mol^−1^). The more energy released, the more negative the value is obtained. The phase conversion from Pb‐I toward Pb‐F in [PbI_6‐x_F_x_]^4−^ octahedra in the precursor is thermodynamically favorable. Then, the formed Pb‐F bonds enable the perovskite crystallization starting at the surface of corner‐sharing [PbI_6‐x_F_x_]^4−^ octahedra due to the similar framework of [PbF_6_]^4‐^ and [PbI_6_]^4−^. It is believed that the fluoride‐terminated [PbI_6‐x_F_x_]^4−^ octahedra are more easily converted to the [PbI_6_]^4−^ framework than the intercalated PbI_2_‐DMSO or face‐sharing δ‐FAPbI_3_. As a result, the TFAI‐incorporated film undergoes nearly complete phase transition even at room temperature. In addition, the fluoride‐terminated [PbI_6‐x_F_x_]^4−^ framework also serves as the nucleus and facilitate homogeneous nucleation which is favorable for high‐quality crystal growth.^[^ ^45^ ^]^ Upon annealing (stage III), a complete phase transition and an enlarged grain size are observed in the TFAI‐incorporated perovskite film.

**Figure 2:**
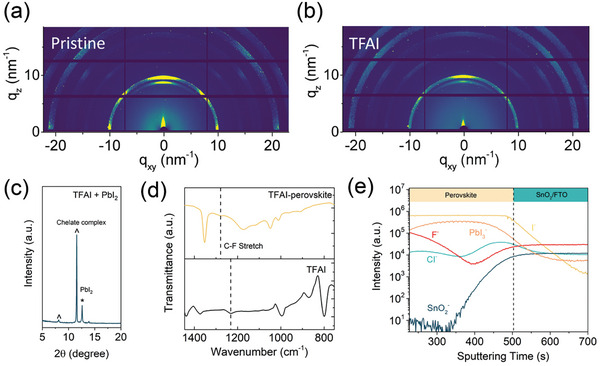
Films feature characteristics. Grazing‐incident wide‐angle X‐ray scattering (GIWAXS) 2D patterns of a) pristine and b) TFAI‐incorporated perovskite film. c) XRD pattern from TFAI + PbI_2_ mixture dissolved in DMF/DMSO. The as‐deposited film is annealed at 120°C for 10 min. d) Fourier transform infrared (FTIR) spectroscopy of TFAI raw film and TFAI‐incorporated perovskite films. e) Time‐of‐flight secondary ion mass spectrometry (ToF‐SIMS) of TFAI‐incorporated perovskite films.

The Grazing‐incident wide‐angle X‐ray scattering (GIWAXS) technique is a powerful tool for investigating the film crystallinity and phase information. Synchrotron measurements were performed at a fixed grazing incidence of 0.2°. **Figure** **2**a,b show the 2D GIWAXS patterns of pristine and TFAI‐incorporated perovskite film. The perovskite films are well‐crystallized and ‐textured along the *q_z_* direction, where *q_z_* represents the out‐of‐plane scattering vector. The specific signals at *q* = ≈10 nm^−1^ and 8.9 nm^−1^ correspond to the (001) plane of cubic α‐FAPbI_3_ and (001) plane of hexagonal PbI_2_, respectively.^[^ ^5^, ^52^ ^]^ The films exhibit both the Debye scatter ring, representing random orientation, and semi‐Bragg spots, representing oriented grain, indicating competition between isotropic and anisotropic crystallization near the surfaces.^[^ ^53^ ^]^ The TFAI‐incorporated film shows the suppressed PbI_2_ phase, which is attributed to the coordination between TFAI and perovskite at GBs.

As limited amount of TFAI introduced and preferred distribution of TFAI at GBs, there is no peak regarding the TFAI‐perovskite production identified in the GIWAXS results. Therefore, the film from the precursor containing only TFAI and PbI_2_ is prepared to collect the XRD pattern (Figure 2c). After annealing, the resultant shows strong signals located at 8.13° and 11.58° with respect to the coordinated complex through Pb‐F chelation. Fourier transform infrared (FTIR) spectroscopy was used to further verify these results (Figure 2d; Figure S6, Supporting Information). The raw TFAI film, pristine perovskite films, and TFAI‐incorporated perovskite films were exposed to irradiation ranging from 700–4000 cm^−1^. The bands at 3269 and 3402 cm^−1^ are assigned to N‐H stretch vibration. The characteristic absorption peak of C═N stretch and N‐H bending vibration appeared at 1704 and 1606 cm^−1^, respectively. The C‐F stretch located at 1230 cm^−1^ from the C‐F terminal group in the TFAI is identified. The peak shifts to 1276 cm^−1^ after introducing TFAI into the perovskite film. Halogen hydrocarbons, especially chloride and fluoride are highly sensitive to the chemical environment of the neighboring groups. This distinct shift implies the coordination of the Pb‐F chelation in the TFAI‐incorporated film.

Elemental distribution was evaluated using time‐of‐flight secondary ion mass spectrometry (ToF‐SIMS). Cs and Bi ion guns were used to exfoliate and analyze the film in the negative ion mode. The depth profile of the TFAI‐incorporated perovskite film is shown in Figure 2e and Figure S7a,b (Supporting Information). Molecular fingerprint fragments are selected to distinguish the perovskite and SnO_2_/FTO interface by detecting the I^−^/PbI_3_^−^ and SnO_2_ signals. It was found that F^−^ representing TFAI has a gradient distribution from the surface to the bottom in the perovskite film. Additionally, we observed an inhomogeneous distribution of F^−^ signal from the 2D mapping of perovskite layer (Figure S7c, Supporting Information). The location of TFAI was further confirmed through energy dispersive X‐ray spectroscopy (EDX) measurements. The corresponding SEM images are captured at 20 kV accelerated electron beam as shown in Figure S8a–c (Supporting Information). It's found that the F atoms at the specific signal of 0.68 keV have a higher atomic ratio at perovskite GBs in TFAI‐incorporated perovskite films (Figure S8d, Supporting Information). The reduced I (Figure S8e, Supporting Information) and Pb (Figure S8f, Supporting Information) atoms ratio at GBs suggest that the ions are immobilized toward GBs and that there is a reduction in PbI_2_ at GBs. Therefore, TFAI is considered to be fixed at GBs in the perovskite film.^[^ ^28^ ^]^

### 2.2 GBs Passivation Mechanism

**Figure 3:**
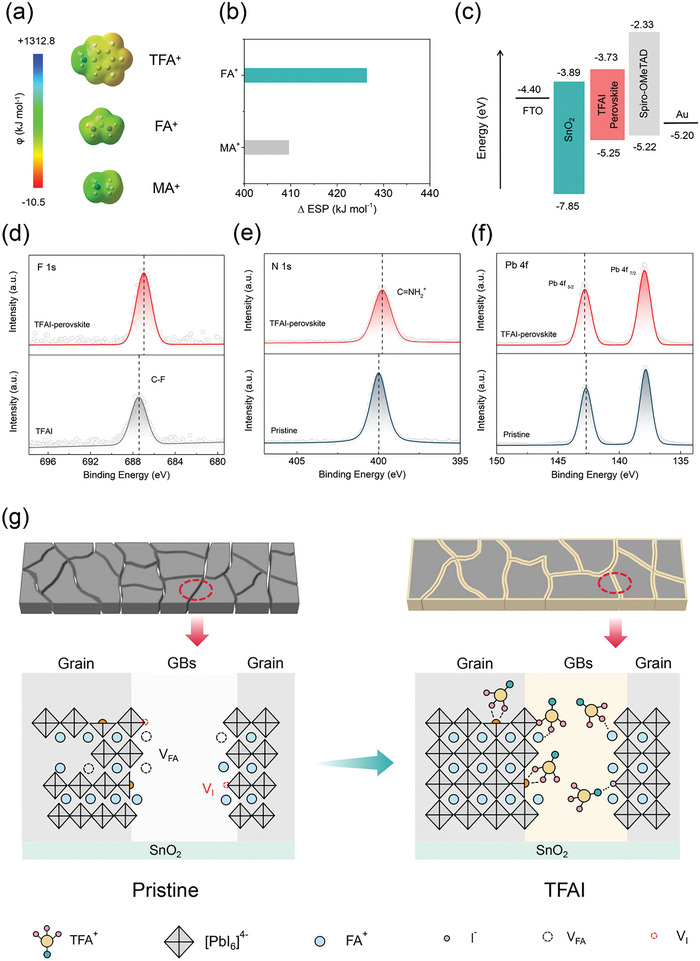
Manipulated chemical bonding at GBs. a) Electrostatic surface potential distribution of TFAI+, FA+ and MA+ through DTF calculation. b) The electrostatic surface potential difference (∆ESP) between the electron‐rich part of TFA+ and the electron‐deficient part of FA+ and MA+. c) Band alignment of FTO/SnO2/Perovskite/Spiro‐OMeTAD/Au derived from absorption and UPS spectra. XPS analysis of d) F 1s orbitals of raw TFAI and TFAI 1% films and e) N 1s and f) Pb 4f orbitals of pristine and TFAI 1% films. g) Schematic diagram of GBs passivation. The TFAI chelated with uncoordinated Pb^2+^ suppress the generation of non‐radiative recombination centers, while the bonds between TFAI and FA^+^, I^−^ prevent perovskite from producing vacancy defects and block the ion migration at GBs.

We further gain insights into the GBs passivation mechanism. Since the great amount of MACl additive (35 mol%) helping for crystallization is introduced into the perovskite recipe, the chemical activity between TFA^+^, FA^+^, and MA^+^ should be checked first. **Figure** **3**a shows the electrostatic surface potential (ESP) distribution of TFA^+^, FA^+^, and MA^+^ as revealed by the DFT simulation. The red regions indicate electron‐rich areas, while the blue regions indicate electron‐deficient areas. In the case of TFA^+^, the negative ESP of 210.0 kJ mol^−1^ is localized around the 3, 4, 5‐substituted fluoride atoms due to their strong electronegativity, while the positive ESP is concentrated around the amino group. For FA^+^ and MA^+^, the electron‐deficient parts are located around the nitrogen atom. To understand the TFA^+^ interaction between FA^+^ and MA^+^, We calculated the difference in electrostatic surface potential (∆ESP) between the electron‐rich parts of TFA^+^ (fluorine) and the electron‐deficient part of FA^+^ and MA^+^ (nitrogen) as shown in Figure 3b. The ∆ESP of TFA^+^‐FA^+^ is much higher than that of TFA^+^‐MA^+^, which allows for favorable interaction between TFA^+^ and FA^+^.

We note that the dipole moment of TFA^+^ is estimated to be 13.2 Debye, which is much higher than that of FA^+^ (0.21 Debye) and MA^+^ (2.29 Debye) due to the presence of three C‐F groups owning powerful electron‐withdrawing capabilities. Therefore, the strong electric field produced at GBs may shift the energy level of perovskite. Ultraviolet photoelectron spectroscopy (UPS) is employed to monitor the highest occupied molecular orbital (HOMO) and lowest unoccupied molecular orbital (LUMO) of perovskite in the vacuum‐aligned case of the device (Figure 3c; Figure S9, Supporting Information). The HOMO and LUMO levels of pristine perovskite are at −5.37 eV and −3.85 eV, respectively. After incorporating TFAI, there is a relevant shift to higher levels of −5.25 eV and −3.73 eV. The improved band alignment benefits hole transport, while electrons are blocked at GBs preventing the carrier recombination.

X‐ray photoelectron spectroscopy (XPS) analysis was applied to investigate the interaction in the TFAI‐incorporated perovskite film. We have predicted that fluoride components have a high affinity to the perovskite terminal. Figure 3d shows the F 1s signal of raw TFAI and TFAI‐incorporated perovskite film. The binding energy is shifted from 687.36 eV (raw TFAI) to 686.95 eV (TFAI‐incorporated perovskite) indicating a strong chemical connection of Pb‐F chelated coordinate complex. In the pristine sample, the peak at 399.97 eV in N 1s (Figure 3e) is identified as the C═NH_2_^+^ signal of FA^+^. After incorporating, the TFAI peak shifts to lower binding energy by 0.21 eV implying the formation C═NH_2_^+^∙∙∙F hydrogen bonding, which is consistent with the ∆ESP result. The C 1s spectra also confirm FA^+^‐TFA^+^ interaction by decoupling the signals into C═NH_2_^+^ (FA^+^) and standard C‐C peaks (Figure S10a, Supporting Information). All the data are calibrated by standard C‐C peaks before analysis. There is no signal belonging to metallic Pb^0^ is observed in either of the pristine and TFAI‐incorporated perovskite films (Figure 3f). This is attributed to the high‐quality crystallization in both films. The spin‐orbital splitting peaks of Pb 4f 5/2 and Pb 4f 7/2 in TFAI‐incorporated perovskite shift to higher binding energy by 0.12 eV, which is attributed to strong electron‐withdrawing capability of F in TFAI,. Besides. The I 3d spectra (Figure S10b, Supporting Information) suggest that the NH_3_^+^∙∙∙I hydrogen bonding between TFA^+^ and I^−^ is suggested responsible for the shift of I 3d 3/2 and I 3d 5/2.

Therefore, we propose the GBs passivation mechanism through TFAI incorporation as illustrated in Figure 3g. The uneven growth of GBs in the pristine sample, caused by anisotropic crystallization, result in creation of intense dangling bonds. These unsaturated atoms at GBs subsequently form point defects, which commonly act as trap‐states and nonradiative recombination centers at GBs of perovskite due to the localized chemical environment and asymmetric fields.^[^ ^54^, ^55^ ^]^ The point defect traps at perovskite GBs are mainly composed of negative charged FA vacancy (V_FA_)^[^ ^56^, ^57^ ^]^ and positive charged I vacancy (V_I_)^[^ ^58^ ^]^ with low formation energy at perovskite GBs. Upon introducing TFAI into the precursor, the passivator ligands interact with the edge of the perovskite crystals during the grain growth. As annealing time is prolonged, the ligands are mainly fixed at GBs due to the limited reactivity to bulk perovskite induce isotropic crystallization. Therefore, it is expected that TFAI will effectively passivate the GBs in‐situ after annealing. First, the localized TFAI interacts with uncoordinated Pb^2+^ by forming Pb‐F chelate complex. The fixed Pb^2+^ prevents perovskite from producing non‐radiative recombination centers.^[^ ^48^ ^]^ Second, the fluoride terminal group in TFAI serving as the electron donor enables the formation of the FA^+^‐TFA^+^ bonding to suppress the V_FA_ at GBs.^[^ ^28^ ^]^ Third, TFAI immobilizes the ion migration through the GBs channels by anchoring the I^−^ or compensating the I^−^ loss during the annealing process.^[^ ^36^ ^]^

### 2.3 Regulated Crystallization with GBs Passivation Toward Efficient PSCs

The incidence‐dependent GIWAXS technique was used to investigate the effects of GBs passivation in perovskite films (Figure S11, Supporting Information). The azimuth integration results are presented in Figure S12a,b (Supporting Information) and Figure S13a,b (Supporting Information). The pristine sample shows the preferred orientation (PO) of the α‐FAPbI_3_ (001) plane along 90 degree. It is caused by the MACl additive, which is a wildly applied to enlarge the crystal size but also induces the preferred anisotropic orientation in the pristine sample.^[^ ^18^, ^59^ ^]^

However, the uneven growth of crystals from the PO of (001) α‐FAPbI_3_ plane results in the undesired defects and strain formation at GBs.^[^ ^47^, ^60^ ^]^ Near the surface of the pristine sample, a slightly weakened growth orientation of 0.1° is observed compared to the bulk value of 0.4° (Figure S12a, Supporting Information). This is due to the initiation of anisotropic nucleation at the air‐liquid interface inward of the bulk.^[^ ^61^ ^]^ Therefore, It is crucial to regulate the PO. In the TFAI‐incorporated sample, the PO of (001) perovskite plane is suppressed in the bulk (0.4°), while the integrated signal near 90 degree is slightly increased at the surface (0.1°) (Figure S12b, Supporting Information). This phenomenon is considered to be due to isotropic growth, which helps mitigate asymmetric fields and release strain. We further confirm the isotropic growth by increasing the TFAI incorporation ratio up to 5%. The perovskite film is dominated by random crystallization, both at the surface and in the bulk (Figure S13a,b, Supporting Information).

The incidence‐dependent 1D patterns are acquired from the corresponding 2D GIWAXS results. The PbI_2_ signal nearly disappears near the surface of TFAI‐incorporated film due to the gradient distribution of TFAI interacting with PbI_2_ at GBs (Figure S12c, Supporting Information), as penetration depth decreases from ≈390 nm (0.4°) to 9 nm (0.1°) (Figure S14, Supporting Information). Additionally, we observed a slight shift of the α‐FAPbI_3_ (001) peak in the pristine sample toward a larger *q_xy_* vector from the bulk to the surface. It's suspected that the microstrain localized at the surface is related to the lattice strain induced by the difference in the thermal expansion coefficient^[^ ^62^ ^]^ and lattice mismatch between perovskite and SnO_2_/FTO.^[^ ^63^, ^64^ ^]^ Upon increasing the TFAI to 5%, The (001) peak remains consistently located at ≈9.96 nm^−1^, indicating the alleviation of residual strain in the film (Figure S13c, Supporting Information). Multiple pin holes were found at the interface between the pristine perovskite and the SnO_2_/FTO substrate, while the TFAI‐incorporated sample shows a very dense and compact structure along the cross‐section as shown in Figure S15 (Supporting Information).

**Figure 4:**
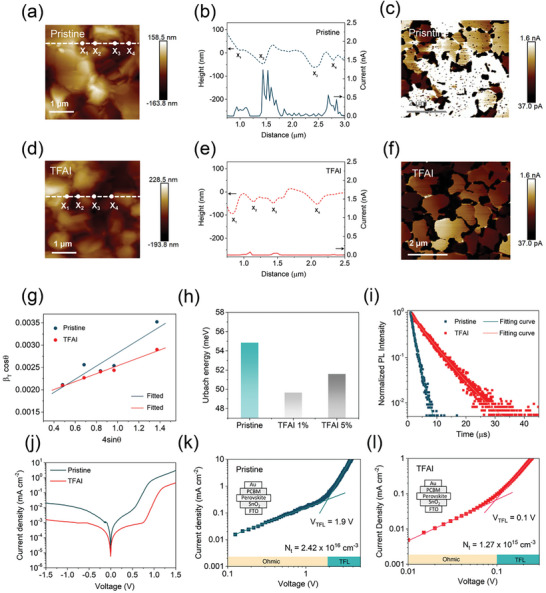
Defects passivation in perovskite films. The C‐AFM height mapping (a,d) and current‐height 1D profiles (b,e) of pristine and TFAI‐incorporated samples at 1.0 V bias. The spots from X_1_ to X_4_ represent the GBs in line. The current mapping of pristine c) and TFAI‐incorporated f) samples at 2.0 V bias. g) Williamson‐Hall plots of the pristine and TFAI‐incorporated perovskite films. h) Urbach energy calculation of the pristine and TFAI 1% and TFAI 5% films. i) TRPL of the pristine and TFAI‐incorporated perovskite films. j) The dark currents of pristine and TFAI‐incorporated PSCs. The SCLC characteristics of the k) pristine and i) TFAI‐incorporated electron‐only devices.

Conductive atomic force microscopy (C‐AFM) is applied to study the carrier properties of perovskite films at both grains and GBs under dark condition. **Figure** **4**a,d and Figure S16 (Supporting Information) show the height and current mapping of pristine and TFAI‐incorporated films measured at 1.0 V bias. The dash lines along the scan vector are plotted in the images, where white spots X_1_ to X_4_ represent marked GBs. In the pristine sample, the current sharply arises at GBs X_2_ (1.2 nA) (Figure 4b). Following the incorporation of TFAI, the average current at GBs decreased significantly from 900 pA to 43 pA (Figure 4e). In addition, we applied a higher bias of 2.0 V to probe the electrical characteristics of the perovskite grains. The current mapping images are presented in Figure 4c,f. The leakage current at GBs become more pronounced under higher bias in the pristine sample. Moreover, we noted that the dark current in the grains of the pristine perovskite was becoming more significant (average 2.1 nA) as presented in the white regions (Figure 4c). In contrast, the TFAI‐incorporated film shows a suppressed average recombination current (300 pA) in the grains. The reduced dark current under external bias suggest a reduction in recombination centers and mitigated ion migration caused by the suppressed vacancies in the TFAI‐incorporated films.^[^ ^64^, ^65^ ^]^

The XRD results reveal the crystallographic information of the perovskite as depicted in Figure S17a (Supporting Information). The XRD patterns collected for both pristine and TFAI‐incorporated perovskite films reveal a preferred crystallization along (001). It is observed that the peak intensity decreases with an increase in TFAI incorporation ratio. Therefore, we conducted a full‐angle integrated 1D GIWAXS along vector *q* at the surface (0.1°) and in the bulk (0.4°), as shown in Figure S17b,c (Supporting Information). The result indicates that the intensity of (001) α‐FAPbI_3_ peaks is comparable. Therefore, we conclude that the less preferred orientation in XRD is due to isotropic crystallization in the TFAI‐based perovskite, rather than an issue of crystallinity. We chose to analyze the full width at half maximum (FWHM) information of (002) and (012) planes due to their intense diffraction and high multiplicative factor, which provide insights into the reliable symmetry structure.^[^ ^54^ ^]^ The full width at half maximum (FWHM) of the (002) plane initially decreases after the introduction of 1% TFAI, but then increases as TFAI is increased to 5% (Figure S18a, Supporting Information). In contrast, the FWHM of the (012) plane continues to decrease even when TFAI is increased to 5%. It is suspected that the reduction in residual strain may affect the microstructure of the perovskite.

We further carry out the Williamson‐Hall plots of the perovskite films to confirm the idea as the isotropic growth is favorable for releasing the microstrain (*ε*). The peak broadening from the strain effect was evaluated using selected planes such as (001), (011), (012), and (111), as shown in Figure 4g. The pristine perovskite holds positive *ε* values of 1.47 × 10^−3^, indicating that the film remains under tensile strain.^[^ ^66^ ^]^ As the uneven growth is suppressed, the *ε* decreases to 8.90 × 10^−4^ in the TFAI‐incorporated film. When the TFAI content comes to 5%, the residual strain is further reduced resulting in the *ε* of 5.00 × 10^−4^ (Figure S18b,c, Supporting Information).

Strain reduction in perovskite benefits the suppression of defects generated in the films.^[^ ^11^ ^]^ These defects produce an energy band tail deep down into the band gap and form intermediate states. Materials with poor crystallinity, disordered structures, and unpassivated grain boundaries typically cause the band tail to appear, which can be quantified by evaluating the Urbach energy (*E_u_*).^[^ ^67^ ^]^ A reduced *E_u_* in the perovskite film indicates the well‐grown grains and suppressed *V_oc_* loss. Figure 4h compares the *E_u_* of the perovskite films derived from the refined absorption spectra (Figure S19a, Supporting Information). The *E_u_* of pristine, TFAI 1%, and TFAI 5% perovskite films are 54.85, 49.66, and 51.59 meV, respectively (Figure S19b, Supporting Information). The *E_u_* demonstrates a similar variation tendency with microstrain implying a conjugated relationship between GBs/strain and trap states.

The carrier lifetime is studied by the time‐resolved photoluminescence spectra of perovskite films deposited on the glass substrates. A biexponential decay model was applied to fit the TRPL curves as shown in Figure 4i.^[^ ^31^ ^]^ The fast decay time constants (*τ_1_*) related to the radiative recombination of pristine and TFAI‐incorporated perovskite films are 0.88 and 2.13 µs, while the slow decay time constants (*τ_2_*) due to the trap‐assisted recombination are 1.81 and 5.69 µs, respectively. The prolonged lifetime is ascribed to the fully grown and well passivated perovskite layer with TFAI.^[^ ^68^ ^]^ The incorporation of TFAI in the film has resulted in an increase in the average lifetime (*t_ave_*) from 1.21 to 5.28 µs, indicating a high‐quality film. It is worth noting that even with an over‐introduction of 5% TFAI, the *t_ave_* only experienced a small reduction to 4.60 µs (Figure S20, Supporting Information). These results are further supported by the 2D PL mapping (Figure S21, Supporting Information). The carriers generated by the photo were mainly extracted at the interface between ITO and TFAI‐perovskite, rather than being recombined in the pristine sample. Upon removing the ITO layer, the PL intensity of TFAI 1% significantly increased on the glass substrate, indicating suppressed nonradiative recombination in the bulk. However, as the TFAI concentration increases to 5%, the PL intensity on glass decreases, possibly due to the intervened crystallization.

The measured dark currents measured in TFAI‐incorporated PSCs show a decrease of one magnitude in reverse saturation current compared with pristine PSCs (Figure 4j). We then conducted the space‐charge limited current (SCLC) from electron‐only devices with FTO/SnO_2_/Perovskite/PCBM/Au structure to analyze the carrier behavior. The Ohmic (linear area) and trap‐filled limited (nonlinear area) regions were identified by the kink point of trap‐filled limited voltage (*V_TFL_*). After carrying out the trap‐states density calculation, It is found that the density of trap states in the TFAI‐incorporated device (1.27 × 1015 cm^−3^) is one order of magnitude lower than that in the pristine device (2.42 × 1016 cm^−3^) (Figure 4k,l). The light‐intensity dependent *J_sc_* and *V_oc_* are fitted in Figure S22a,b (Supporting Information). Both of the curves show well‐fitted linearity in the power law relationship *J_sc_* ∝*I* ^α^, where *I* is the light intensity. The ideal factor (*n*) from the illuminated diode equation is estimated to be 1.75 kT/q for the pristine PSCs. It has been found that the *V_oc_* loss at low light intensity regimes is suppressed after TFAI incorporation. The reduced n of 1.11 kT/q indicates the reduced nonradiative recombination. The built‐in potential (*V_bi_*) is an important factor in assessing the carrier separation under illumination. A high *V_bi_* contributes to the suppressed recombination at GBs.^[^ ^69^ ^]^ By performing calculations through the Mott‐Schottky equation, we found that the *V_bi_* increased from 0.87 V (pristine) to 1.00 V (TFAI‐incorporated) (Figure S23a, Supporting Information).

**Figure 5:**
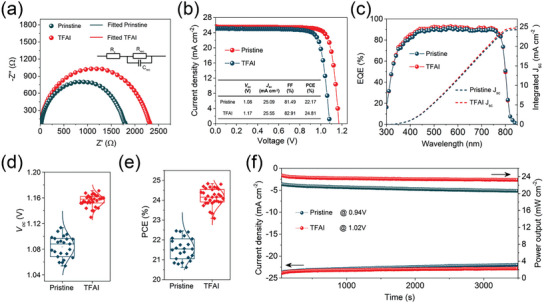
PSCs performance a) The Nyquist plots of pristine and TFAI‐incorporated PSCs from EIS measurement (Inset: The equivalent circuit model). b) The J‐V curves of the champion device for pristine and TFAI‐incorporated PSCs. c) The EQE measurements and integrated current density of pristine and TFAI‐incorporated PSCs. The performance parameter distribution of d) *V_oc_* and e) PCE for pristine and TFAI‐incorporated PSCs. f) the steady power outputs (SPO) of pristine and TFAI‐incorporated PSCs under constant applied bias near the maximum power output points.

The electrochemical impedance spectroscopy (EIS) of PSCs was measured at the external DC bias equaling their built‐in potentials under the dark condition to lower the carrier density (**Figure** **5**a). This measurement benefits the study of the charge transport and recombination properties. The equivalent circuit is shown as an inset. The TFAI‐incorporated PSC shows an increase in charge recombination resistance (R_rec_) from 1780 Ω (pristine) to 2290 Ω indicating the reduced trap‐assisted charge recombination in the device at low frequency regimes. Additionally, the series resistance (R_s_) slightly shifted from 10.5Ω (pristine) to 7.2 Ω (TFAI‐incorporated) PSCs at high‐frequency regimes, which is attributed to high‐quality crystallization after introducing TFAI.

We further fabricated PSCs with the regular planar structure of FTO/SnO_2_/Perovskite/Spiro‐OMeTAD/Au. To achieve record efficiency, interface modification is now considered a standard strategy. It should be noted that the TFAI‐incorporated champion PSC, without any pre‐buried interface or upper capping layer, mainly reinforced *V_oc_* from 1.08 to 1.17 V due to the effectively regulated grains and passivated GBs (Figure 5b). The J‐V curves show a slight increase in *J_sc_* from 25.09 mA cm^−2^ in the pristine sample to 25.55 mA cm^−2^ in the TFAI‐incorporated sample. The champion PCE of 24.81% is attained after incorporation, while the best performance of pristine PSCs is 22.17% as shown in **Table** **1**. After conducting external quantum efficiency (EQE) characterization on TFAI‐incorporated PSCs, we also observed an increased integrated *J_sc_* from 24.2 mA cm^−2^ to 24.7 mA cm^−2^ (Figure 5c). This increase is attributed to the improved signal response across the full UV‐Vis spectrum. Previous studies have reported that well‐crystallized perovskite impacts absorbance and, subsequently, photo‐generated current.^[^ ^18^ ^]^ The device hysteresis was evaluated in Figure S23b (Supporting Information). The hysteresis index of the FAI‐incorporated PSCs has been reduced from 9.9% to 2.3% due to the suppressed trap‐assisted recombination.

**Table 1:** Performance parameters of pristine and TFAI‐incorporated PSCs.

Device	Average/Champion	*V_oc_* [V]	*J_sc_* [mA cm^−2^]	FF [%]	PCE [%]	Series resistance [Ω cm^2^]	Shunt resistance [Ω cm^2^]
Pristine	Average	1.08 ± 0.015	24.58 ± 0.26	80.68 ± 0.68	21.48 ± 0.50	3.96 ± 0.52	3615 ± 1431
	Champion	1.09	25.16	81.43	22.45	3.49	4059
TFAI	Average	1.16 ± 0.007	25.22 ± 0.27	82.87 ± 0.44	24.15 ± 0.34	2.78 ± 0.16	9556 ± 631
	Champion	1.17	25.55	82.91	24.81	2.69	9703

Figure 5d,e show the distribution of the *V_oc_* and PCE performance parameters for 55 individual pristine and TFAI‐incorporated PSCs. The average *V_oc_* increased from 1.08 to 1.16 V, while the average PCE improved from 21.48% to 24.15% after TFAI incorporation. We further study the TFAI‐dependent performance distribution from 0% to 5% as shown in Figure S24 (Supporting Information). The *V_oc_* and FF have an up‐down tendency as the TFAI increases. We propose that the over‐involved crystallization by incorporated TFAI causes the *V_oc_* loss after 1%. It is interesting to note that the overloaded TFAI has barely any impact on *J_sc_*, which supports the view that TFAI is primarily located at GBs. The relevant performance is summarized in Table S1 (Supporting Information). The steady‐state power output (SPO) was recorded in Figure 5f. The applied bias is fixed near the maximum power output point (MPP) of PSCs. The TFAI‐incorporated PSC shows slight decay (1.5%) at first 170 s and further maintains its 95.5% initial PCE after 3500 s, while the PCE of pristine PSC keeps decreasing from the beginning to the end, where the performance maintains its 92.8% initial value. The prolonged SPO testing (over 380 h) on both pristine and TFAI‐incorporated PSCs is shown in Figure S25 (Supporting Information). We applied the constant bias near the MPPs (0.93 V for TFAI‐incorporated PSC and 0.86 V for pristine PSC) The TFAI modified PSC with initial PCE of 24.30% maintain almost its 100% initial PCE over 380 h. Although the PCE of pristine PSC show sable at first 1 day, the SPO dramatically decreased after 1.5 day. The pristine PSC only holds less its 89% initial PCE after 380 h.

## 3 Conclusion

We present a film regulation strategy that involves incorporating 3,4,5‐Trifluoroaniline iodide (TFAI) into the perovskite precursor. This approach achieves both crystallization modulation and passivation of grain boundaries through precursor engineering. TFAI ensures complete perovskite phase transition at room temperature and facilitates isotropic crystallization. We found that TFAI spontaneously localizes at grain boundaries and serves as a passivation agent through engineered bonding to the perovskite. The chelation of Pb‐F anchors the uncoordinated Pb^2+^ at GBs, preventing perovskite degradation into PbI_2_. The interaction between FA^+^ and TFA^+^ passivates the vacancy defects. Therefore, TFAI enables reduced dark current in grains and suppresses leakage current at GBs. The *V_oc_* of TFAI‐incorporated PSCs without interface modification improves from 1.08 to 1.17 V, which is one of the highest *V_oc_* among the analogs. The champion device achieved a PCE of 24.81%. The PSCs incorporated with TFAI under a bias of 1.02 V near the MPP exhibit stable operation power output after 380 h without any pre‐processing or encapsulation. This work presents a facial precursor engineering approach that involves co‐worked crystallization modulation and GBs passivation, aimed at upscaling and commercializing PSCs.

## Conflict of Interest

The authors declare no conflict of interest.

## Author Contributions

Z.J.S. and Y.Q.Z. performed conceptualization. Z.J.S., Y.X.W., and J.W. performed methodology. Y.Y.W., X.Z., and H.L.W. acquired software. Z. J. Shi., X. G. Li., and C. Y. Li. performed formal analysis. Z.J.S., X.F.Y., and L.L.D. performed investigation. A.R.Y., Y.Q. Z., C.X.C., and Y.G.Y. acquired resources. A.R.Y. and Y.Q.Z. performed supervision. Z.T.X., A.R.Y., and Y.Q.Z. performed funding acquisition.

## Supporting information

Supporting Information

## Data Availability

The data that support the findings of this study are available from the corresponding author upon reasonable request.
