# Supplementary material for: Room Temperature Crystallized Phase‐Pure α‐FAPbI3 Perovskite with In‐Situ Grain‐Boundary Passivation
Source: Adv Sci (Weinh). 2024 Mar 19;11(22):2400275. doi: 10.1002/advs.202400275 (PMC11165534; doi:10.1002/advs.202400275)
Supplement: Supplementary file 1 — Supporting Information [file ADVS-11-2400275-s001.pdf]

ADVANCED SCIENCE
Open Access

Supporting Information

for *Adv. Sci.*, DOI 10.1002/advs.202400275

Room Temperature Crystallized Phase-Pure α-$FAPbI_3$ Perovskite with In-Situ Grain-Boundary Passivation

Zejiao Shi, Yaxin Wang, Yanyan Wang, Xiaoguo Li, Xiaofei Yue, Haoliang Wang, Xin Zhang, Liangliang Deng, Chongyuan Li, Jiao Wang, Zuoti Xie, Yinguo Yang, Chunxiao Cong, Anran Yu and Yiqiang Zhan**

Supporting Information

Room Temperature Crystallized Phase-pure α-$FAPbI_3$ Perovskite with In-situ Grain-boundary Passivation

Zejiao Shi, Yaxin Wang, Yanyan Wang, Xiaoguo Li, Xiaofei Yue, Haoliang Wang, Xin Zhang, Liangliang Deng, Chongyuan Li, Jiao Wang, Zuoti Xie, Yinguo Yang, Chunxiao Cong, Anran Yu and Yiqiang Zhan**

Z. J. Shi, Y. X. Wang, Y. Y. Wang, X. G. Li, X. F. Yue, H. L. Wang, X. Zhang, L. L. Deng, C. Y. Li, Prof. J. Wang, Prof. C. X. Cong, Prof. A. R. Yu and Prof. Y. Q. Zhan

Center for Micro Nano Systems

School of Information Science and Technology (SIST)

Fudan University

Shanghai, 200433, P. R. China

E-mail: aryu@fudan.edu; yqzhan@fudan.edu.cn

Prof Y. G. Yang.

School of Microelectronics

Fudan University

Shanghai, 200433, P. R. China

Prof Z. T. Xie

Department of Materials Science and Engineering

MATEC

Guangdong Technion - Israel Institute of Technology

Shantou, Guangdong, 515063, P. R. China

Methods

Materials:

ITO glasses are customized and patterned by Sunyo Technology Co,. Ltd. SnO_2 dispersed nanoparticle colloid (tin (IV) oxide, 15%) and 3,4,5-Trifluoroaniline, 99% are purchased from Alfa Aesar. Formamidinium iodide (FAI) is purchased from Greatcell Energy. Lead iodide (PbI_2) is purchased from TCI. Methylenediammonium dichloride ($MDACl_2$), Hydroiodic acid (57 wt. % in H_2O), Tris(2-(1Hpyrazol-1-yl)-4-tert-butylpyridine) cobalt(III) tri[bis(trifluoromethane) sulfonimide] (FK 209; Co(III) TFSI salt), Bis(trifluoromethane)sulfonimide lithium salt (Li-TFSI) are purchased from Sigma Aldrich. Methylammonium chloride (MACl) is purchased from Shanghai MatterWin New Materials Co., Ltd. 2,2',7,7'-tetrakis(N, N-dip-methoxyphenylamine)-9,9'-spirobifluorene (Spiro-OMeTAD) is purchased from Xi'an Yuri Solar Co., Ltd. Ethanol, N, N-Dimethylformamide (DMF), Dimethyl sulfoxide (DMSO), Chlorobenzene, isopropanol (IPA), 4-tert-butylpyridine (4-tBP) and acetonitrile (ACN) are purchased from Thermo Fisher. Diethyl ether is purchased from Sinopharm Chemical Reagent Co., Ltd. Phenyl-C61-butyric acid methyl ester ($PC_{61}BM$) is purchased from Nano-C.

Synthesis:

3,4,5-trifluoroaniline iodide (TFAI): TFAI is prepared by mixing 4321 mg 3,4,5-Trifluoroaniline and 8.14 ml hydroiodic acid in 40 ml ethanol, followed by ice bathing and stirring for 3 hours. The brown precipitates is obtained after the solution is dried and filtrated. To purify the product, diethyl ether is used to wash the precipitates and remove the excess ligands three times. After that, The precipitates is fully dissolved into ethanol to recrystallize. The wash and redissolve flow is repeated three times. Finally, the collected white TFAI powder is dried in the vacuum oven at 60°C overnight.

Device fabrication:

The ITO glasses are sequentially cleaned with detergent, deionized (DI) water,

acetone and IPA in the ultrasonic tank for 15 min and then dried by compressed air. After baking at 150°C for 10 min, Oxygen plasma treatment is applied to remove the surface contamination. The SnO_2 layer from the diluted 2 % dispersion colloid is spin-coated on the substrate at 3000 rpm for 30 s followed by annealing at 80°C for 50 min in a vacuum oven. The prepared substrate is then transferred into the N_2-filled glove box after UV-zone treatment for further perovskite layer deposition. The perovskite solution prepare from FAI (1.4 M), PbI_2 (1.4 M), MACl (0.5 M), $MDACl_2$ (0.05 M) and TFAI (0, 0.5 mol%, 1 mol%, 2 mol% and 5 mol%) in anhydrous DMF/DMSO (8:1 (v:v)) is deposited on the SnO_2 substrate at 1000 rpm for 10 s and 5000 rpm for 10 s. 1 ml anti-solvent chlorobenzene is drop on the spinning perovskite film with or without TFAI at the last 20 s in the second step. The as-deposited perovskite film is sequentially annealed at 150 °C for 15 min for complete crystallization. Spiro-OMeTAD hole transport solution (72.3 mg ml^{-1}) in 1.5 ml chlorobenzene is prepared by mixing 25 μl Li-TFSI (520 mg ml^{-1} in ACN), 27.5 μl Co(III) TFSI (300 mg ml^{-1} in ACN) and 37.5 μl 4-tBP additives. The doped Spiro-OMeTAD solution is spin-coated on the perovskite layer at 3000 rpm for 30 s. For the SCLC testing, PCBM solution (15 mg ml^{-1} in chlorobenzene) is deposited on the film at 1000 rpm for 60 s and 5000 rpm 2s to form a hole blocking layer. Finally, 85 nm gold electrode is thermally evaporated on the top of the device at 10^{-5} Pa to complete the fabrication process.

Characterization：

The top view and cross-section view of Scanning Electron Microscopy (SEM) images are captured by ZEISS GeminiSEM 450 in the field-emission scanning model with an accelerated 2 kV electron beam. Energy Dispersive X-ray spectroscopy (EDX) analyses are conducted by Phenom ProX with 20 kV accelerating bias. The Grazing-Incident Wide-Angle X-ray Scattering (GIWAXS) patterns of perovskite films are collected at Shanghai Synchrotron Radiation Facility (SSRF) using a 1.24 Å X-ray resource. The films are illuminated by X-rays from the incident angle from 0.1

to 0.4°. The 2D and 1D GIWAXS patterns are analyzed by FIT2D along the scattering vector q. The XRD patterns of perovskite films are examined from an X-ray diffractometer (Rigaku SmartLab 3 kW) with a monochromatic CuKα (λ = 1.54 Å) X-ray source. The Fourier Transform Infrared (FTIR) spectroscopy spectra of perovskite films are measured by Thermo Scientific Nicolet iS20 with DTGS KBr detector and KBr beam splitter. The signal of samples and backgrounds are scanned over 32 times from 400 to 4000 cm^{-1}. The Time-of-Flight Secondary Ion Mass Spectrometry (ToF-SIMS) of perovskite films is characterized by ToF-SIMS 5-100 instrument (IONTOF GmbH). The Depth profile and 3D reconstruction render are conducted using a negative ion model. Cs ion gun is applied to remove the component layer by layer at 1KeV in a 200 × 200 μm sputter area. Bi_3^{++} with 30 keV energy is used to analyze the component in a 50 × 50 μm acquisition area. Ultraviolet Photoelectron Spectroscopy (UPS) spectra are measured by Thermo Scientific Escalab Xi+. -5 V bias is applied on the film during testing. The energy step is fixed at 0.05 eV. X-ray Photoelectron Spectroscopy (XPS) measurements are carried out using Thermo Fisher Scientific K-Alpha+. The irradiation spot (200 × 200 μm) is focused by an Al K Alpha source gun. The analyzer mode is selected as CAE: Pass Energy 80.0 eV with an energy step of 0.1 eV. Conductive Atomic Force Microscopy (C-AFM) images of perovskite films are conducted from Bruker instrument. Surface topographies, current mapping images and 1D patterns along scan direction are analyzed by NanoScopeAnalysis software. Absorption spectra of perovskite films are measured using an ultraviolet spectrophotometer (SHIMADZU UV-1750) from 550 nm to 850 nm. Steady-state PL spectra are carried out by an F20-UV thin-film analyzer (FILMETRICS) with 405 nm laser excitation. PL mapping of perovskite films on glass and FTO/glass substrates are examined by MicroRaman spectroscopy (WITEC Alpha300R) with a 532 nm excitation source. Voltage-Capacitance (C-V) plots and Electrochemical Impedance Spectroscopy (EIS) of the devices are measured by an electrochemical workstation Zennium-pro (Zahner). For the C-V testing, the AC frequency is set at 10 kHz with an amplitude bias of 10 mV under dark. For the EIS measurement, the applied DC bias is fixed at V_{bi} with an AC amplitude of 50 mV

scanning from 100 Hz to 3MHz under dark. The corresponding data is fitted by ZView software. Time-resolved photoluminescence (TRPL) spectra are recorded by the FLS1000 Edinburgh Spectrometer. The films are excited using a 475 nm laser source. The J-V measurements of the devices are performed using a Keithley 2600 Source Meter under AM 1.5G (100 mW cm^{-2}) standard irradiation generated by Newport Sol3A ClassAAA simulator with a 450 Watt Xenon lamp. An NREL-produced Si reference solar cell is used to calibrate the light output intensity before testing. External quantum efficiency (EQE) measurement is attained by the QE-R system (Enlitech) with a 75-Watt Xenon lamp. The monochromatic light is calibrated by a NIST-traceable Si detector from 300-850 nm before testing. The molecular calculations of electrostatics potential (ϕ) are performed on GaussView and Gaussian 09W software.

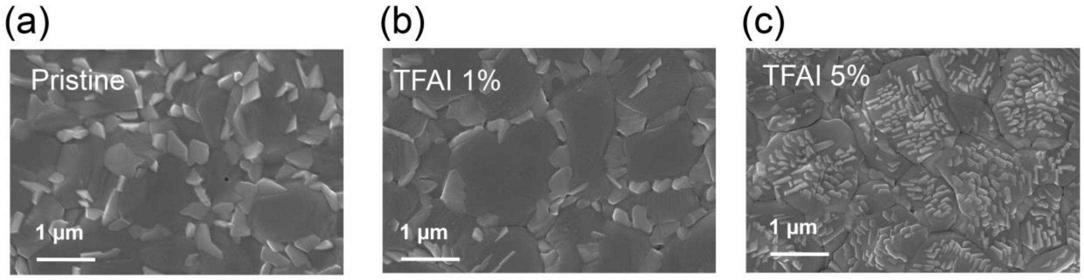

Figure S1. Scanning electron microscopy (SEM) images of top-viewed perovskite surface morphology involving (a) 0% (pristine), (b) 1% and (c) 5% TFAI.

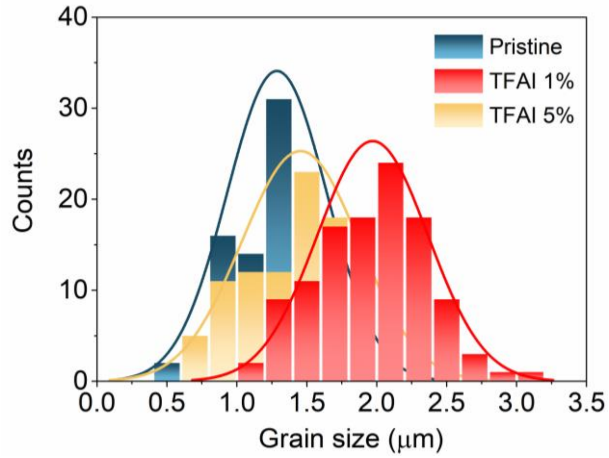

Figure S2. Statistical distributions of perovskite grain size with and w/o TFAI incorporation.

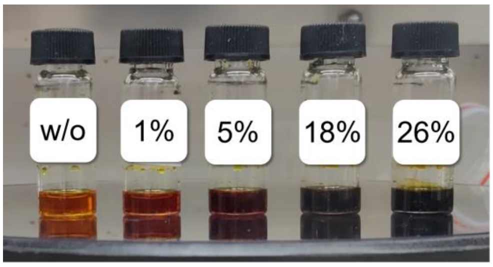

Figure S3. Photographs of prepared perovskite precursor w/o and with 1%-26% TFAI incorporation.

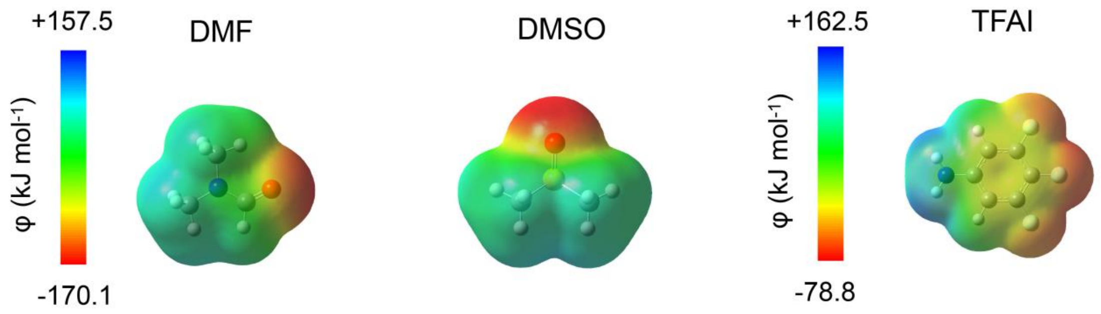

Figure S4. Electrostatic surface potential (ESP) distribution of DMF, DMSO and TFAI.

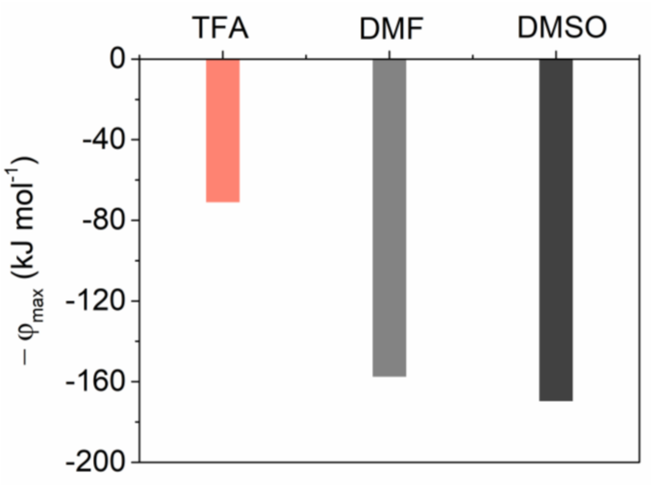

Figure S5. The negative ESP ($-\phi_{max}$) of TFA, DMF and DMSO.

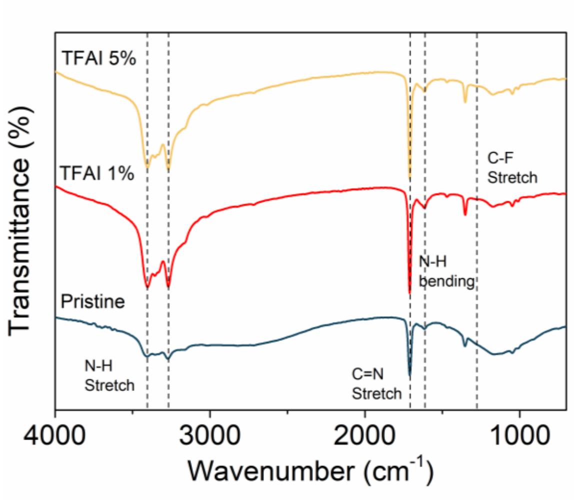

Figure S6. FTIR spectra of pristine and TFAI-incorporated perovskite films.

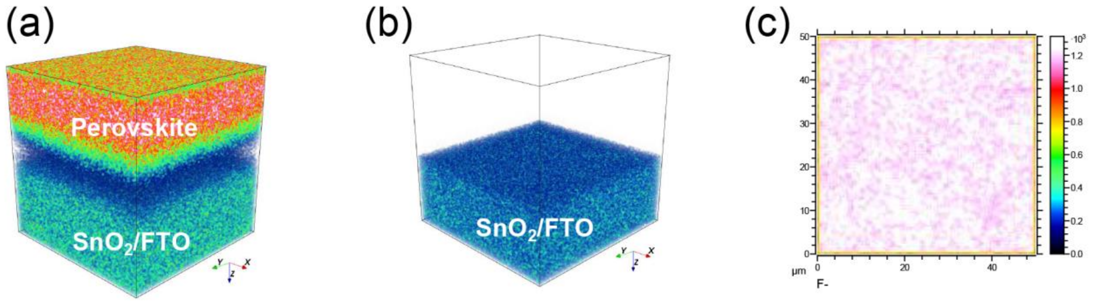

Figure S7. ToF-SIMS of 3D render reconstruction of depth profile from (a) F^- and (b) SnO_2 signal. (c) 2D mapping of F^- in perovskite layer.

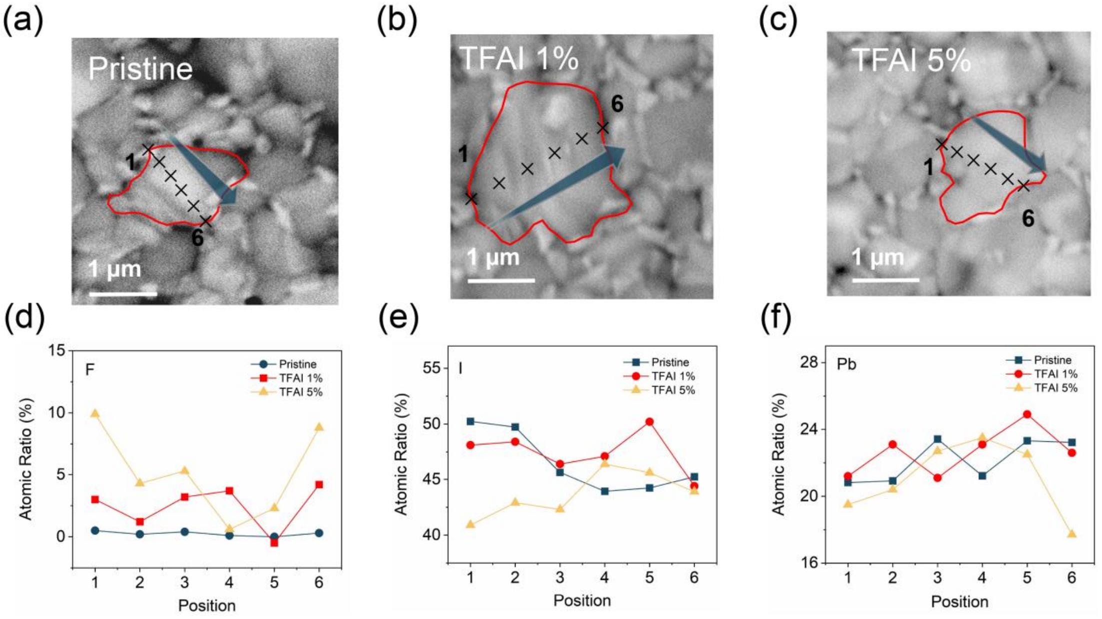

Figure S8. The SEM images of (a) pristine (b) TFAI 1% and (c) TFAI 5% perovskite films captured with an accelerated electron beam at 20 kV. The red curves represent the perovskite GBs. Spots 1 to 3 represent the positions from GB to grain. The spots 4 to 6 represent the positions from grain to GB. EDX results of (d) pristine (e) TFAI 1% and (f) TFAI 5% films measured at corresponding positions 1 to 6. Spot model is applied to obtain the elemental information at points of SEM images.

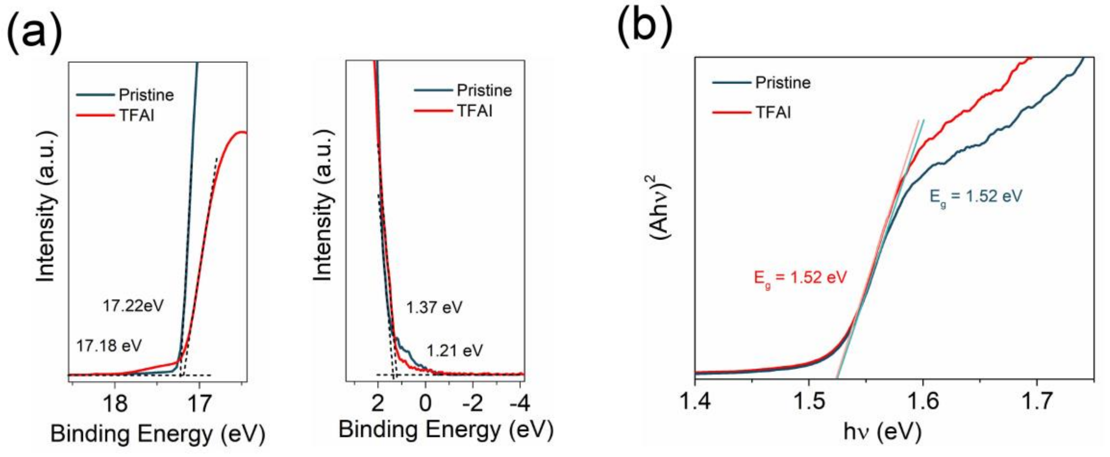

Figure S9. (a) UPS spectra of pristine and TFAI-incorporated perovskite films at secondary electron cut-off $E_{cut\text{-}off}$ (left) and onset E_i (right). (b) Tauc plots of pristine and TFAI-incorporated perovskite films.

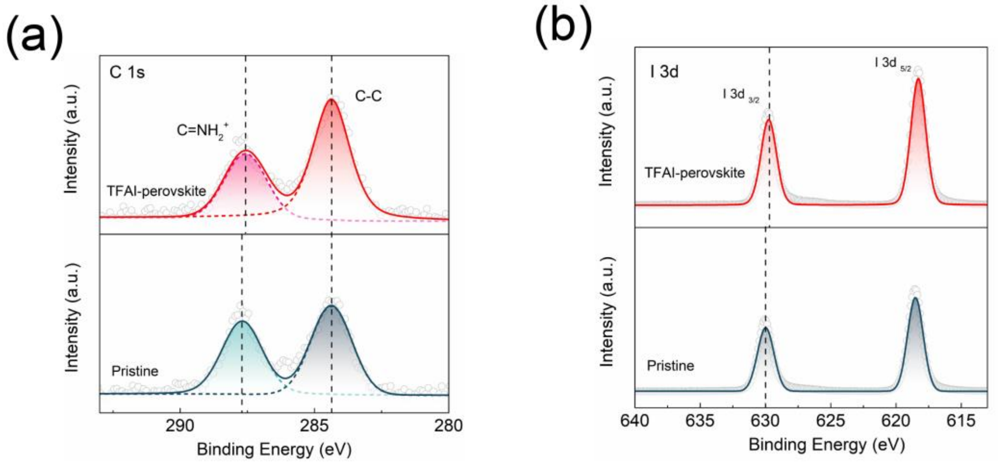

Figure S10. XPS spectra of (a) C1s and (b) I 3d of pristine and TFAI-incorporated perovskite films.

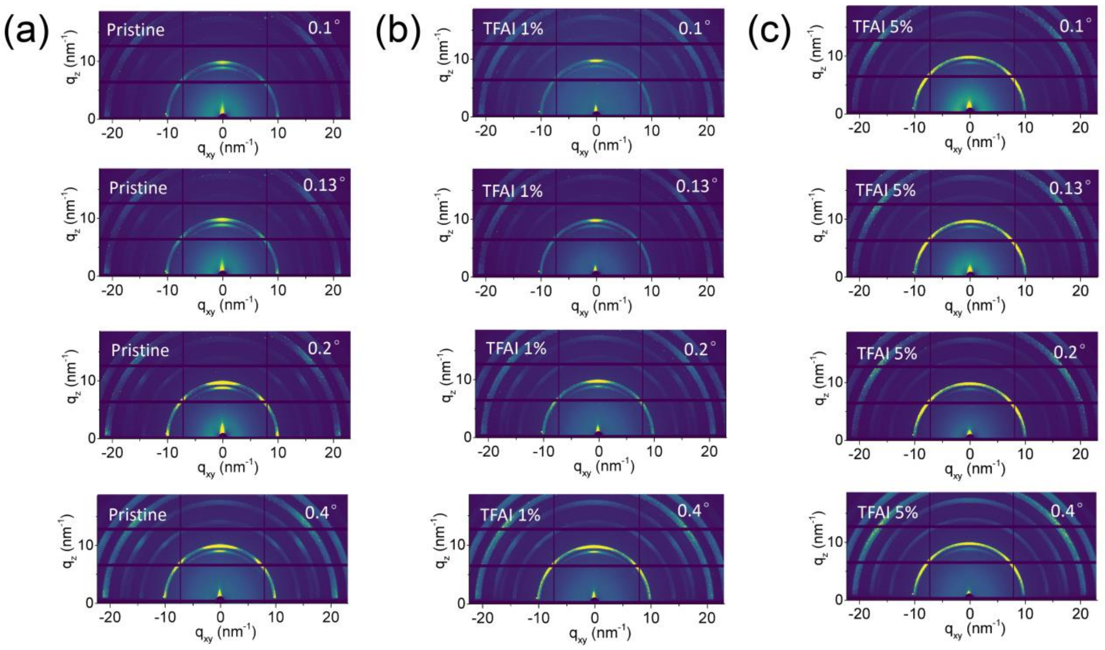

Figure S11. The incidence-dependent 2D GIWAXS patterns of (a) pristine, (b) TFAI 1% and (c) TFAI 5% perovskite films.

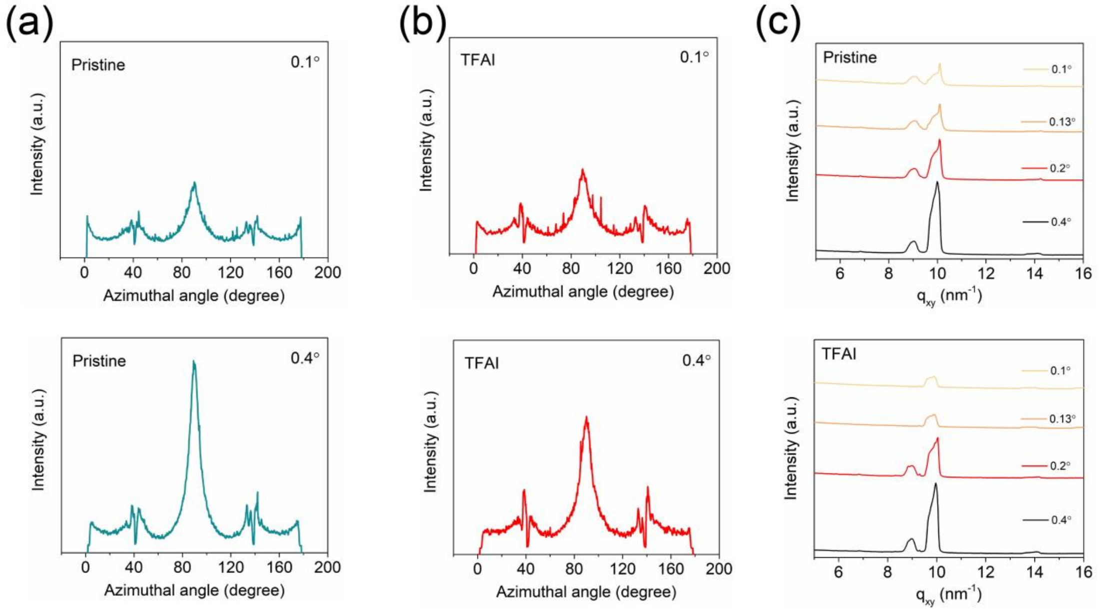

Figure S12. Integrated azimuth angle for (a) pristine and (b) TFAI-incorporated perovskite films at $q_z \approx 10$ nm^{-1} (The incidence is fixed at 0.1° (upper) and 0.4° (bottom), respectively). (c) The incidence-dependent 1D GIWAXS patterns of pristine (upper) and TFAI incorporated (bottom) films along the out-of-plane vector.

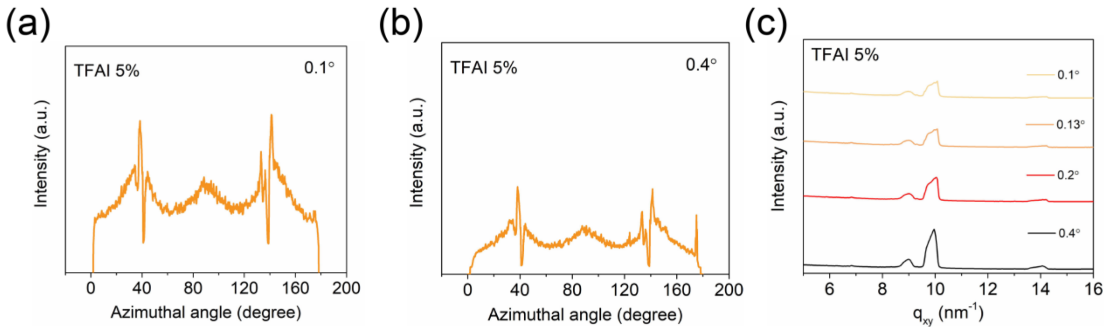

Figure S13. (a) Integrated azimuth angle for TFAI 5% perovskite film collected at (a) 0.1º and (b) 0.4º. (c) The incidence-dependent 1D GWAXS pattern of TFAI 5% perovskite film along out-of-plane vector.

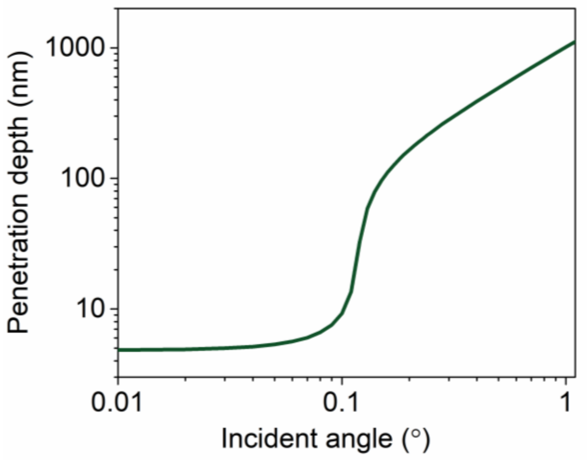

Figure S14. the incidence dependent penetration depth of X ray applied in GIWAXS technique.

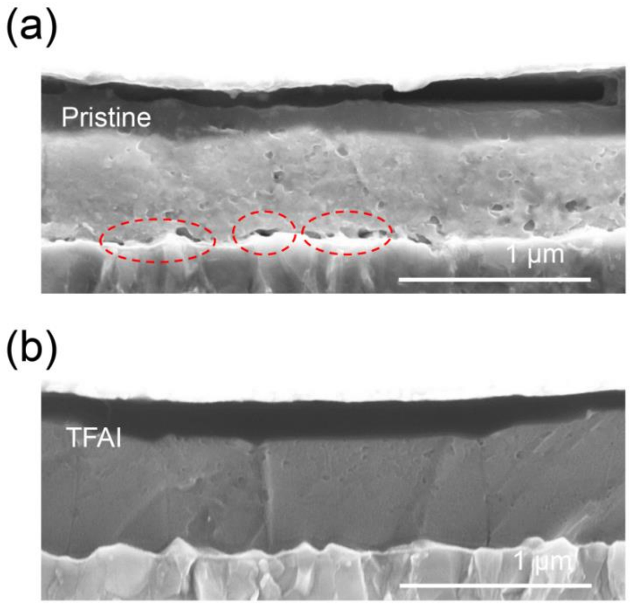

Figure S15. cross-section SEM images of pristine and TFAI-incorporated perovskite films. The red dash circles show the pin holes lying at the interface between perovskite and SnO_2/FTO substrates.

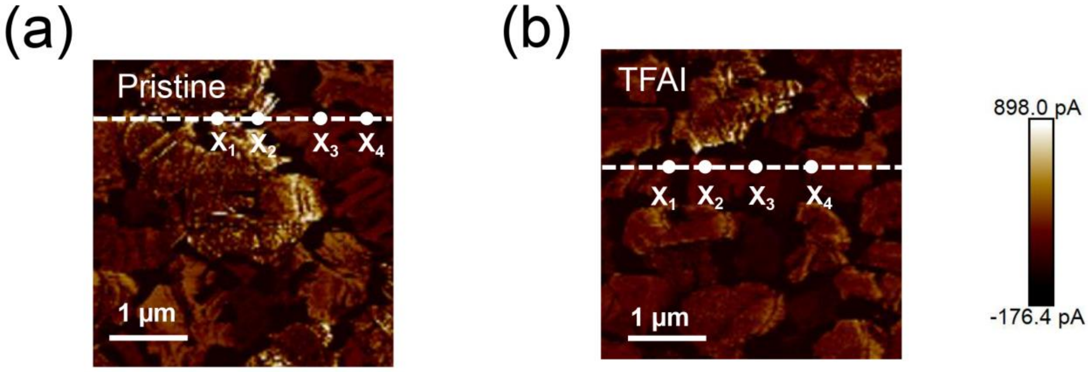

Figure S16. The C-AFM current mapping of pristine and TFAI-incorporated perovskite films at 1.0 V bias. The spots from X_1 to X_4 represent the GBs in line.

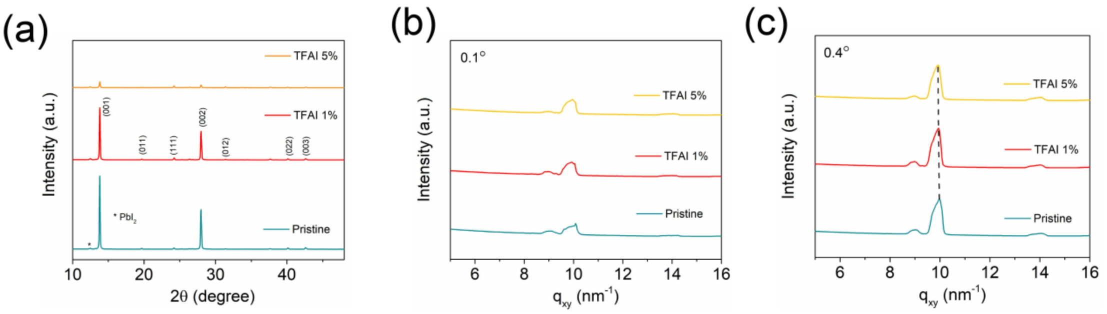

Figure S17. XRD (a) and full-angle integrated 1D GIWAXS patterns at 0.1° (b) and 0.4° (c) incidence .

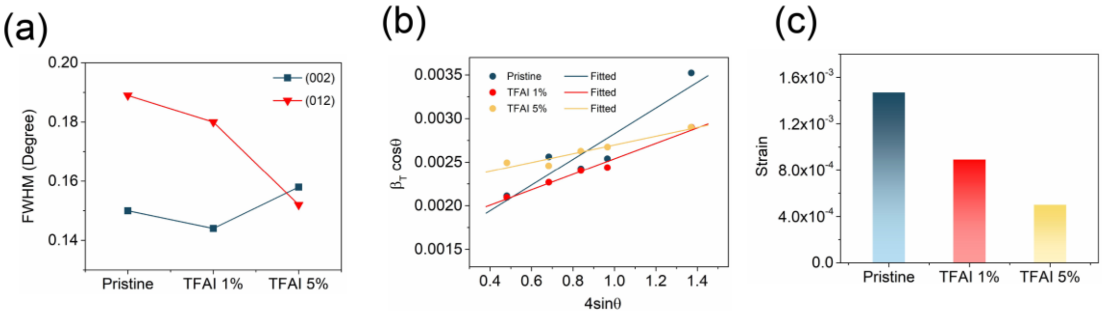

Figure S18. (a) (001) and (012) FWHM of pristine, TFAI 1% and TFAI 5% perovskite films.

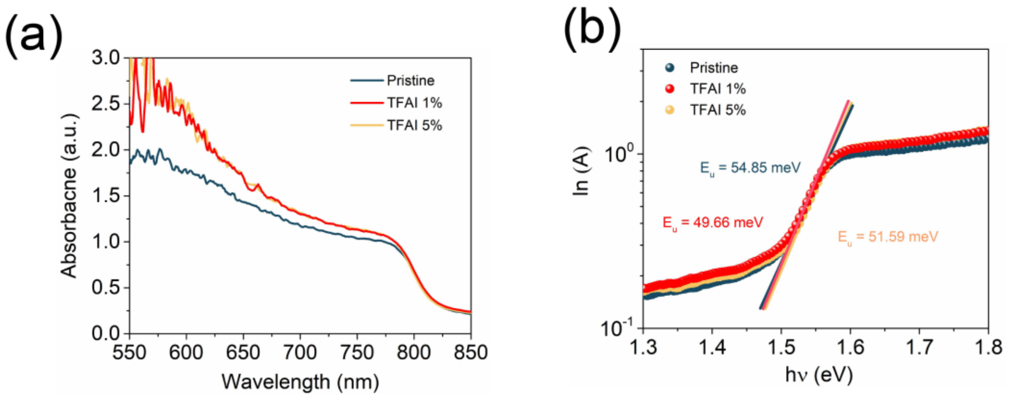

Figure S19. (a) Absorption spectra and (b) absorbance logarithm versus photo energy of pristine, TFAI 1% and TFAI 5% perovskite films.

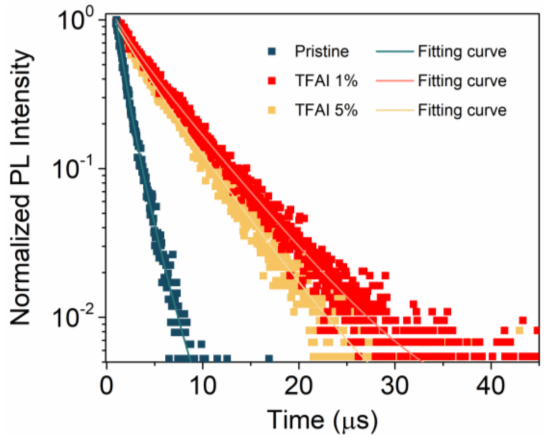

Figure S20. TRPL spectra of pristine, TFAI 1% and TFAI 5% perovskite films.

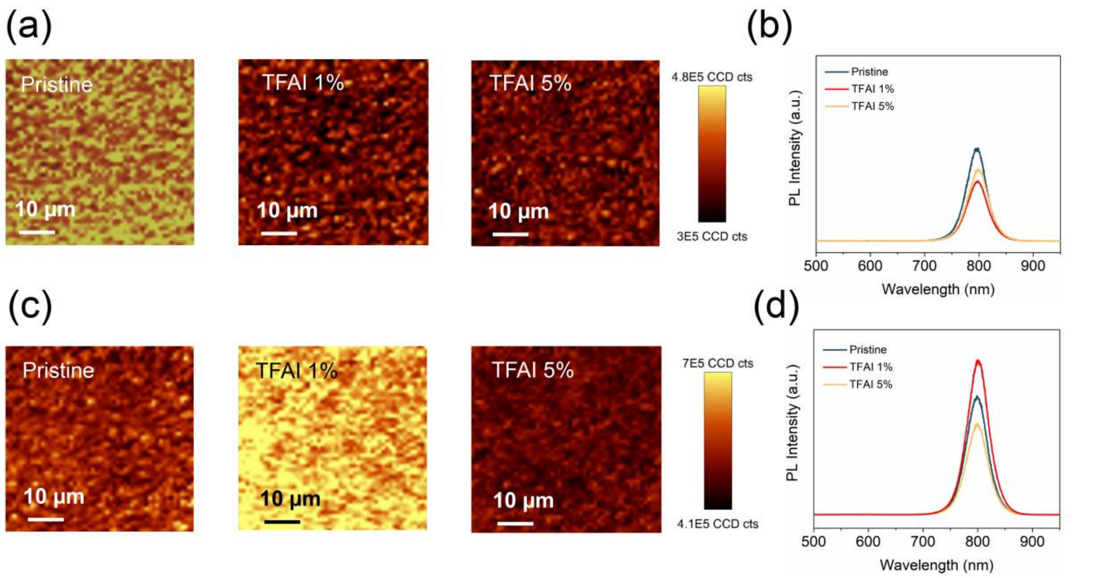

Figure S21. The PL mapping of pristine, TFAI 1% and TFAI 5% films on (a) ITO and (c) glass substrates. The corresponding PL spectra are plotted on (b) ITO and (d) glass substrates.

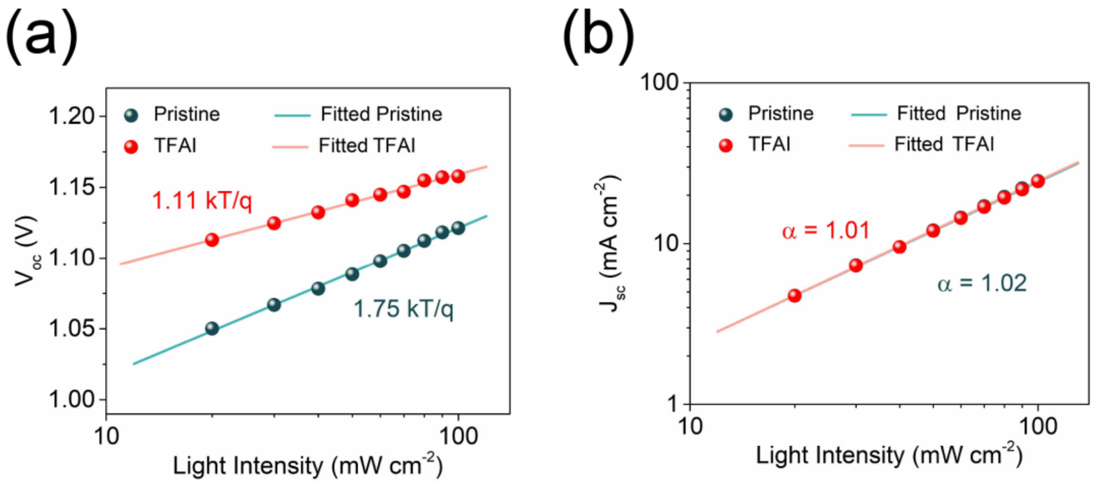

Figure S22. Light-intensity dependent (a) V_{oc} and (b) J_{sc} of pristine and TFAI-incorporated PSCs.

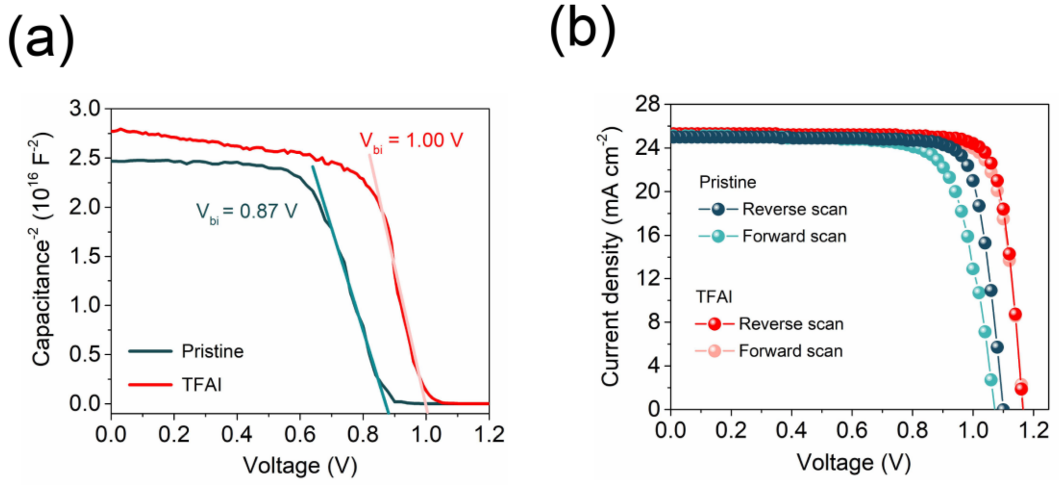

Figure S23. (a) Mott-Schottky plots of the pristine and TFAI-incorporated perovskite PSCs. (b) The J-V curves of pristine and TFAI-incorporated PSCs under forward and reverse scan directions.

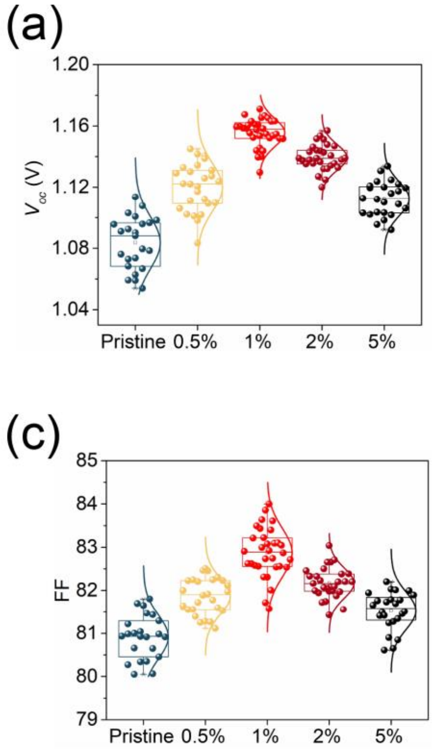

Figure S24. V_{oc}, J_{sc}, FF and PCE performance parameters of pristine, TFAI 0.5%, TFAI 1%, TFAI 2% and TFAI 5% PSCs.

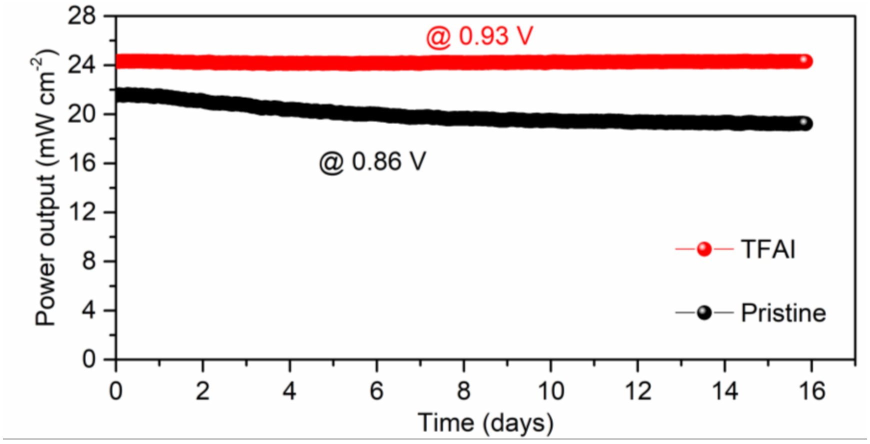

Figure S25. Prolonged SPO test of pristine and TFAI-incorporated PSCs under constant applied bias near the maximum power output points.

Table S1. Performance parameters of pristine and TFAI-incorporated PSCs

Device	Average/Champion	V_{oc} [V]	J_{sc} [mA cm^{-2}]	FF [%]	PCE [%]	Series resistance [Ω cm^2]	Shunt resistance[Ω cm^2]
Pristine	Average	1.08 ± 0.015	24.58 ± 0.26	80.68 ± 0.68	21.48 ± 0.50	3.96 ± 0.52	3615 ± 1431
	Champion	1.09	25.16	81.43	22.45	3.49	4059
TFAI 0.5%	Average	1.11 ± 0.014	24.95 ± 0.30	81.57 ± 0.65	22.67 ± 0.48	3.05 ± 0.33	5124 ± 957
	Champion	1.14	25.31	81.98	23.62	2.80	5385
TFAI 1%	Average	1.16 ± 0.007	25.22 ± 0.27	82.87 ± 0.44	24.15 ± 0.34	2.78 ± 0.16	9556 ± 931
	Champion	1.17	25.55	82.91	24.81	2.69	9703
TFAI 2%	Average	1.14 ± 0.007	25.12 ± 0.27	82.17 ± 0.27	23.52 ± 0.31	2.93 ± 0.25	8711 ± 1027
	Champion	1.14	25.73	82.07	24.15	2.72	9161
TFAI 5%	Average	1.11 ± 0.009	25.11 ± 0.32	81.13 ± 0.87	22.56 ± 0.50	3.03 ± 0.37	7557 ± 1154
	Champion	1.13	25.47	82.20	23.73	3.20	8044

Supplementary Note 1. Energy release of PbI_2 and PbF_4 formation.

To perform the calculation, one free Pb^{2+} ion and one free I^- or F^- ion are bonded together in the internuclear distance r_0. The energy release during this process is defined as $-E_{one}$ [1].

In PbI_2 case, E_{one} regrading the constructed one ion pair (Pb-I) from one Pb^{2+} and one I^- is estimated as follows:

$$E_{one} = k\frac{Q_1 Q_2}{r_0} \qquad \text{Eq. 1}$$

Where k is the electric field constant (2.31 x 10^{-28} J·m), Q_1 and Q_2 is the ion charge and r_0 is the internuclear distance of Pb-I (306 pm)[2]. The calculated E_{one} of PbI_2 is -1.6×10^{-18} J. To further obtain the formation energy of one mole PbI_2 ($E_{(PbI_2)}$), the followed equation is applied:

$$E_{(PbI_2)} = \frac{E_{one}\, P_{(PbI_2)}}{N_A} \qquad \text{Eq. 2}$$

Where $P_{(PbI_2)}$ is the number of Pb-I bonds in PbI_2 and N_A is Avogadro's number. The $E_{(PbI_2)}$ of PbI_2 is estimated to be -1806 kJ mol^{-1}.

In PbF_4 case, the r_0 of Pb-F is affirmed as 219 pm[2]. E_{one} of -2.1×10^{-18} J regrading the constructed one ion pair (Pb-F) from one Pb^{2+} and one F^- is estimated from Eq. 1. The formation energy of PbF_4 ($E_{(PbF_4)}$) is further evaluated as -5056 kJ mol^{-1} by Eq. 2.

Hence, the released energy of PbI_2 and PbF_4 is roughly analyzed from above discussion. Compared to the $E_{(PbI_2)}$, $E_{(PbF_4)}$ possesses more negative value implying the phase conversion from PbI_2 to PbF_4 is thermodynamic permitted. We also search the predicted formation energy of PbF_4 (-1.99 eV/atom) and PbI_2 (-0.95 eV/atom) to support our results[3]. Besides, the Pb-F bond dissociation energy of 3.8 eV is found much higher than that of PbI_2 (2.0 eV) suggesting the thermodynamic stable bond of Pb-F [4, 5].

Supplementary Note 2. Micro strain calculation from Williamson-Hall plots.

The total broadening (β_T) of the XRD peaks is combined by the effects from crystal size (β_D) and micro strain (β_ε):

$$\beta_T = \beta_D + \beta_\varepsilon \qquad \text{Eq. 3}$$

The β_D can be derived from the Scherrer equation:

$$D = \frac{K\lambda}{\beta_D \cos\theta} \qquad \text{Eq. 4}$$

Where K is the shape factor, λ is the wavelength of incident X ray (1.54 Å), D is the crystal size derived from SEM statistics distribution and θ is the radians peak location.

The β_ε can be given as follows:

$$\beta_\varepsilon = 4\,\varepsilon \tan\theta \qquad \text{Eq. 5}$$

Where ε is the micros strain and θ is the radians peak location.

The ε is consequently obtained by combine the eq.4 and eq. 5 into the following:

$$\beta_T \cos\theta = 4\varepsilon \sin\theta + \frac{K\lambda}{D}$$

Supplementary Note 3. Urbach energy calculation.

The Urbach energy of perovskite films is derived by the following equation:

$$A = A_0\, exp\,(\frac{hv}{E_u}) \qquad \text{Eq. 6}$$

Where A is the absorbance, A_0 is a constant, hv is the photo energy. Therefore the band tail Eu can be calculated from the slope of the logarithm A versus hv.

Supplementary Note 4. TRPL carrier life fitting.

The decay curves are fitted using biexponential functions as follows:

$$y = A_1\, exp\,(\frac{-t}{\tau_1}) + A_2\, exp\,(\frac{-t}{\tau_2}) \qquad \text{Eq. 7}$$

Where τ_1 is fast decay time constant,τ_2 is slow decay time constant, t is the measured time, A_1 and A_2 are relevant coefficients.

The average life time are estimated by:

$$t_{ave} = \frac{\sum A_i {\tau_i}^2}{\sum A_i \tau_i} \qquad \text{Eq. 8}$$

Supplementary Note 5. Mott-Schottky derived built-in potential.

The built-in potential is carried out from :

$$\frac{1}{C^2} = (\frac{2}{\varepsilon\varepsilon_0 q A^2 N})\,(V_{bi} - V) \qquad \text{Eq. 9}$$

Where C is the capacitance, ε is the vacuum permittivity, ε_0 is the relative permittivity of $FAPbI_3$, q is elementary charge, A is the active area, N is the carrier density and V is applied bias.

Supplementary Note 6. SCLC calculation.

The density of trap states is calculated as the follows:

$$N_t = \frac{2\varepsilon\varepsilon_0 V_{TFL}}{qL^2} \quad \text{Eq. 10}$$

Where N_t is the density of trap states, ε is the vacuum permittivity, ε_0 is the relative permittivity of $FAPbI_3$, V_{TFL} is the trap-filled limited voltage, q is elementary charge and the L is the perovskite thickness.

References

[1] R. Fan, Q. Song, Z. Huang, Y. Ma, M. Xiao, X. Huang, H. Zai, J. Kang, H. Xie, Y. Gao, L. Wang, Y. Zhang, L. Wang, F. Wang, X. Zhang, W. Zhou, N. Li, X. Wang, Y. Bai, G. Liu, Q. Chen, L. Wang, H. Zhou, *Angew. Chem. Int. Ed.* **2023**, *62*, e202303176.

[2] R. D. Shannon, *Acta. Cryst.* **1976**, *A32*, 751.

[3] Https://Next-Gen.Materialsproject.Org.

[4] L. E. Lehner, S. Demchyshyn, K. Frank, A. Minenkov, D. J. Kubicki, H. Sun, B. Hailegnaw, C. Putz, F. Mayr, M. Cobet, G. Hesser, W. Schöfberger, N. S. Sariciftci, M. C. Scharber, B. Nickel, M. Kaltenbrunner, *Adv. Mater.* **2023**, *35*, 2208061.

[5] Y. Luo, *Comprehensive Handbook Of Chemical Bond Energies*, Crc Press, Boca Raton, Fl, Usa **2007**.
